# Intermarriage; or the Mode in Which, and the Causes Why, Beauty, Health, and Intellect, Result from Unions, and Deformity, Disease, and Insanity, from Others: Illustrated by Delineations of the Structure and Forms, and Descriptions of Functions and Capacities, Which Each Parent, in Any Pair, Bestows on Children—in Conforming with Certain Natural Laws, and by an Account of Corresponding Effects in the Breeding of Animals

**Published:** 1852-01

**Authors:** 


ART. XI.—*Intermarriage; or the mode in which, and the causes why, Beauty, Health, and Intellect, result from unions, and Deformity, Disease, and Insanity, from others: illustrated by delineations of the Structure and Forms, and descriptions of Functions and Capacities, which each parent, in any pair, bestows on Children—in conformity with certain natural laws, and by an account of corresponding effects in the Breeding of Animals.* With eight illustrative drawings. By ALEXANDER WALKER. Philadelphia: Lindsay & Blakiston. 1851.

We do not think that Mr. Walker has philosophical knowledge sufficient to enable him to make the best possible work on intermarriage, but he has collected a vast fund of useful facts, which abler minds than Mr. Walker's will shape to valuable ends. In the present condition of society, it can scarcely be expected that any lessons on intermarriage will be productive of much benefit. It is a subject upon which few men, and fewer women, think it worth their while to bestow much study, on the points elaborated by Mr. Walker. The great object of the great mass of the two classes concerned in marriage is first, money; second, position in society; third, convenience. The improvement of the race, the health and vigor of the physical frames of offspring, and the powers of intellection that may be imparted by parents, do not often enter into the intentions of new fledged love. But the subjects of which Mr. Walker treats are of vast and momentous concern, and demand the rigid investigation of every thinking mind. There is an immense amount of, what is called, moral and mental philosophy, taught in the colleges, for which the valuable lessons of Mr. Walker's book might be substituted, greatly to the advantage of society. Brown University has set a noble example in deserting a portion of the antiquated notions of the past on the subject of time-worn formulas of college education, and in substituting therefor utilitarian views and courses. That institution does not hold that it is essential to the making of a good engineer, geologist, metallurgist, or anything else of the kind, that a man must go through the well-worn stages that end in dubbing him A. B. or A. M. The thanks of the country are due to President Maynard for the stand he has taken in opening the portals of Brown University to usefulness and the diffusion of knowledge that is the more real because it is practical.

But, to return to Mr. Walker's book on intermarriage, we think it is filled with a great variety of useful matter that can be made useful to the physiologist. The

[i.e. WAYLAND]

evils of society are deep-rooted, and look firm in the superstructure, but truth is ever fresh and ever vigorous, and error must finally yield. The wonders of change are the crowning glories of the first half of the nineteenth century. Since the beginning of the current century changes have occurred in society, in commerce, science, the mechanic arts, and in all the details of the social state, that amount to the creation of a new era. The thorough adoption and practice of Mr. Walker's principles of intermarriage would not be a greater departure from "that which is old and vanishing away," than the changes we find in a vast proportion of the concerns of the social state.

Mr. Walker's book has been before the public for some years, and has commanded a profound attention. It well deserves the study of society, and we commend it to such of our readers as have not yet perused it, as a book replete with interesting and valuable instruction. The present edition is published in beautiful style by Lindsay & Blakiston. B.

